# Energy Consumption of Young Military Working Dogs in Pre-Training in Germany

**DOI:** 10.3390/ani10101753

**Published:** 2020-09-26

**Authors:** Wencke Schäfer, Julia Hankel

**Affiliations:** 1 Bundeswehr School of Dog Handling, German Armed Forces, 56766 Ulmen, Germany; wenckeschaefer@bundeswehr.org; 2 Institute for Animal Nutrition, University of Veterinary Medicine Hanover Foundation, 30173 Hannover, Germany

**Keywords:** energy requirements, working dog, Malinois, thermoneutral zone, activity level

## Abstract

**Simple Summary:**

Appropriate energy supply adjusted to the requirements of military working dogs at pre-training is a basic prerequisite for working dogs, so they are fully able to exercise. Therefore, twenty Belgian Shepherds var. Malinois were accompanied for four weeks during pre-training as military working dogs, and the amount of energy intake was evaluated. Changes in body constitution were assessed by measuring body weight and shoulder height, as well as thickness of subcutaneous fat tissue, and of Musculus sacrocaudalis dorsalis lateralis (MSCDL). Energy intake amounted to, on average, 244 kcal/kg body weight (BW)^0.75^ daily. Changes to body constitution (increases in body weight, shoulder height, and thickness of the MSCDL, as well as a reduction of the thickness of the subcutaneous fat tissue lying on the MSCDL) might be attributed to training-induced muscle growth and physiological growth in size. In addition to training intensity, influences of ambient temperature and physiological body growth seemed to be important contributing factors in the supply of energy for military working dogs (MWDs) during pre-training.

**Abstract:**

Many factors have specific effects, in varying degrees of intensity, on the metabolic energy requirements of working dogs. Appropriate energy supply adjusted to the requirements of military working dogs at pre-training is a basic prerequisite for working dogs, so they are fully able to exercise. Therefore, more knowledge obtained under standardized conditions would be advantageous. Twenty intact Belgian Shepherds var. Malinois at the age of 12 months were accompanied for four weeks during pre-training (odour detection, obedience training, and protection work) as military working dogs (MWDs) in Germany (ambient temperature about 7.8 °C), and the amount of energy intake was evaluated. To assess changes in body constitution, body weight and shoulder height were measured, and the thickness of subcutaneous fat tissue (SCF), and of Musculus sacrocaudalis dorsalis lateralis (MSCDL), were recorded via ultrasound. Energy intake amounted to, on average, 244 ± 34 kcal/kg body weight (BW)^0.75^ daily. Increases in body weight, shoulder height, and thickness of the MSCDL, as well as a reduction of SCF thickness lying on the MSCDL, were observed. Changes of body constitution might be attributed to training-induced muscle growth and physiological growth in size. In addition to training intensity, influences of ambient temperature and physiological body growth seemed to be important contributing factors in the supply of energy for MWDs during pre-training.

## 1. Introduction

A minimal amount of energy is needed to ensure homeostasis. This amount of energy is described as the basal metabolic rate and indicates the energy needed for metabolic processes in a lying position, awake state, after food digestion is complete, in a thermoneutral ambient temperature without further activity [1]. The so-called maintenance energy requirement (MER) goes beyond the basal metabolic rate. MER is defined as the energy required for thermoregulation, spontaneous activity, and moderate exercise, and can be influenced by factors, such as breed, age, husbandry, body weight, activity level, and thermoregulation [1]. Strenuous exercise and environmental temperature have an effect on energy requirements to varying extents [1]. Appropriate energy supply adjusted to the requirements of military working dogs during pre-training is a basic prerequisite for working dogs, so they are fully able to exercise. For adult dogs at maintenance (laboratory kennel dogs or active dogs), the daily metabolizable energy requirements are generally estimated to be approximately 130 kcal/kg body weight (BW)^0.75^ [1]. When kept in a domestic environment, with little stimulus and opportunity to exercise, daily metabolizable energy requirements are lower, from 95 kcal/kg BW^0.75^ [1] up to 0.410 ± 0.121 MJ/kg BW^0.75^, which corresponds to about 98 ± 29 kcal/kg BW^0.75^ [2]. Values above the average requirements of 130 kcal/kg BW^0.75^ are found for young adult laboratory and active pet dogs, Great Danes, and terriers (140 to 200 kcal/kg BW^0.75^) [1]. Requirements described for hunting dogs amount to 240 kcal/kg BW^0.75^ or for sled dogs pulling heavy loads to 270 kcal/kg BW^0.75^ [1]. Exceptional cases are sled dogs during races, showing kcal requirements of about 1050 kcal/kg BW^0.75^ [1] or 8995 kcal/dog/day up to 13,779 kcal/dog/day [3]. Further studies have demonstrated as well increased energy requirements for working dogs in general (167 kcal/kg BW^0.75^) compared to conventionally kept pet dogs (124 kcal/kg BW^0.75^) [4]. This has also been shown for, similarly, highly active sporting and functional dogs, such as sled dogs and hunting dogs [4]. While energy consumption of agility dogs in North America do not greatly exceed daily metabolizable energy requirements of pet dogs, being about 106 kcal/kg BW^0.75^ per day [5]. Average energy consumption of working dogs trained in odour detection, explosive detection, and human detection, amounted to 136 ± 38 kcal/kg BW^0.75^ [6]. With regard to military working dogs in Germany, however, no specific representative data could be obtained with regard to their specific energy requirements. Metabolic processes can be influenced and altered negatively because of inappropriate energy supply. Under energy deficiency, the ability to perform work is impaired and muscle protein will be catabolized for energy, while on the contrary, an excess of energy leads to overweight and obesity, which is linked to a number of diseases [1]. In order to ensure and optimize physical performance, there is a need for concrete knowledge about appropriate energy supply adjusted to the requirements of each military working dog. One method to measure the daily energy requirements of dogs is by food intake [1].

As the conditions of keeping and training military working dogs are uniform, there is a unique opportunity to acquire further data on daily energy consumption of dogs, with elevated activity levels, under standardized methods. The aim of the present study was to accompany 20 intact Belgian Shepherds var. Malinois for four weeks during their pre-training as military working dogs to draw conclusions about an adequate energy supply of military working dogs during this particular period.

## 2. Materials and Methods

### 2.1. Animals and Husbandry

A total of 20, twelve-month-old intact Belgian Shepherd dogs var. Malinois (between 18.3 and 26.6 kg) were accompanied for four weeks during their preliminary training to become military working dogs (MWDs). Keeping and training were carried out under standardized methods. The dogs were kept in single kennels of a professional kennel facility in compliance with German regulations. The individual kennels had an indoor area as well as a roofed and fastened exterior area in front of the building. The exterior area was freely accessible through a hatch. For daily stays in the open air, fenced areas, as outdoor enclosures, were available. The dogs stayed for between 60 and 180 min daily, with an average of 90 min, in the outdoor enclosure. As the data collection took place in the month of January in Rhineland-Palatinate, the dog kennels were heated by underfloor heating in the indoor area, while the exterior area was not heated. In both sections, the floor was levelled and a wooden doghouse was available in the exterior area. The temperatures were measured daily at the same time and recurrently by means of permanently installed temperature sensors (Renkforce^®^ Thermo-/Hygrometer protein) + (9.4 × g fat) + [4.1 × (g N-free extracts + g fibre)]. Apparent digestibility (ad) of energy (ad GE%) was estimated by fibre content in dry matter. Digestible energy (DE) was calculated as follows: DE = GE × ad GE (%)/100, and finally corrected for nitrogen. The daily energy intake was calculated from the amount of food consumed and its energy density.

### 2.2. Parameters

At the time of the study, the dogs had not yet completed their growth in size and were worked in preliminary training to become MWDs. For this reason, an exclusive recording of body weight would not have allowed any conclusions as to whether an increase in weight was solely due to a possible energy oversupply leading to obesity, growth, or muscle gain. In order to determine the physical constitution, the following parameters were therefore documented in addition to the body weight: shoulder height, thickness of the Musculus sacrocaudalis dorsalis lateralis (MSCDL), and thickness of the subcutaneous fat (SCF) lying on it. In order to avoid rater-dependent results, the measurements mentioned were performed under constant conditions by the author as the only investigator. With regard to possible influencing factors, physical activity and ambient temperature were recorded.

**Figure 1:**
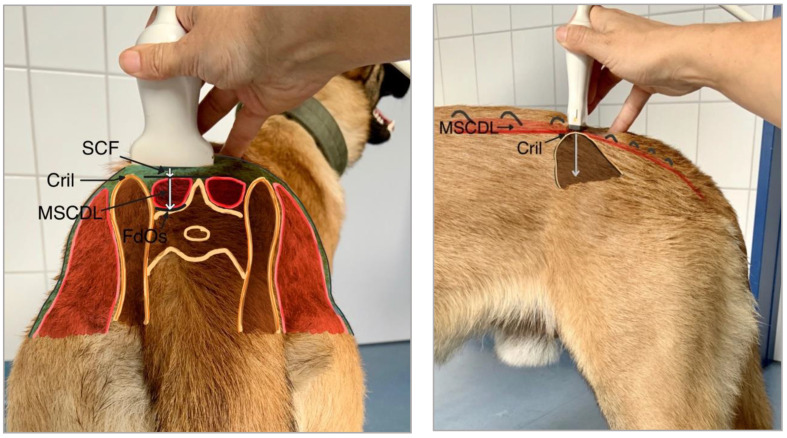
Position of the ultrasound probe on an upright standing dog, Cril: Crista iliaca, FdOs: Facies dorsalis of the Os sacrum, MSCDL: Musculus sacrocaudalis dorsalis lateralis, SCF: subcutaneous fat tissue.

Body weight was recorded on days 1, 7, 14, 21, and 28, and documented in kilograms (kg). The same scale calibrated before the study was used for each measurement to determine body weights. A metal grading scale with millimetre accuracy was used to determine shoulder height. The distance from the highest point of the shoulder to the ground was measured on day 1 and 28 and documented in centimetres (cm). Dorsoventral diameter of the MSCDL and thickness of the on-lying SCF were chosen to be measured. MSCDL, formerly named Musculus sacrococcygeus dorsalis lateralis [7], is the caudomedial elongation of Musculus longissimus [8,9]. A single experienced veterinarian determined the thicknesses of the muscle and subcutaneous fat tissue sonographically. Esaote MyLab™Six with ultrasound probe SL1543 (ESAOTE Biomedica Deutschland GmbH, Köln, Germany) was used. To ensure reliable reproducibility of the measurements, the ultrasound probe was positioned medially of the dorsally most prominent part of the Crista iliaca, at a right angle to the lumbar spine. The position was additionally shaved. In order to reduce possible variability of the data during measurements due to several raters, the same person collected all data (standardized probe position, pressure). Figure 1 shows a schematic diagram of the placement of the ultrasound probe.

**Figure 2:**
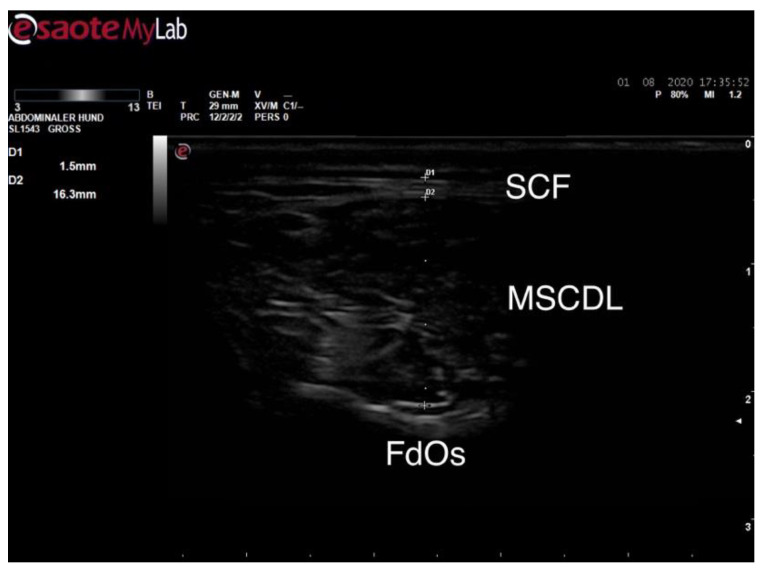
Sonographic documentation of the thickness of the subcutaneous fat tissue (SCF, D1) and Musculus sacrocaudalis dorsalis lateralis (MSCDL, D2), FdOs: Facies dorsalis of the Os sacrum.

After shaving an area of 4 × 2 cm^2^ at the described location, the skin surface was degreased with alcohol and ultrasound gel was applied. Tissue boundaries of subcutaneous fat and muscle tissue between the probe surface and Facies dorsalis of the Os sacrum were detected. On days 1 and 28, the widest thickness of the MSCDL and the SCF, lying on it of each dog, were measured sonographically in an upright and standing position and documented in millimetres (mm). This is shown as an example in Figure 2.

### 2.3. Physical Activity

The study was conducted for four weeks during the pre-training of dogs before their specialized MWD training, i.e., in addition to their own spontaneous activity inside and outside of the kennel, and during the time of care, they were regularly, physically trained to develop (age-appropriate) the necessary physical and mental status, on the basis of which their subsequent specialized training was set up. The possibility described in the literature to determine the level of physical activity by heart rate measurement [10] was not available for the present study. To assess their activity level, the activity performed by the 20 dogs was documented daily. The respective daily training took place on 5 of 7 weekdays in the following activity level categories: long walks on a long leash three times a day for about 30 min or free running for up to 90 min (low activity) to specific service-oriented activities, such as odour detection or obedience training (medium activity). In the training stage of the present group, the dogs were guided for up to 10 min to specifically detect target objects. Such units were conducted up to six times per training unit, according to the level of training. Obedience training increased from the beginning of the training, three times a day for 5 to 10 min to once a day for 20 min. The physically highest demanding training situation (high activity) arose on days with protection work training. Protection work training varied between repeated (up to four times) short units (5 to 10 min) and one-time intensive units (up to 20 min) per training. At least 2 out of 7 days of a week were used for recovery.

### 2.4. Statistical Evaluation

The statistical evaluation was performed with SAS^®^ Enterprise Guide^®^ (Version 7.1). The distribution of the differences was checked using the Shapiro-Wilk test. The differences of the paired observations (shoulder height, body weight, and thickness of MSCDL and SCF between the first and last day of the experiment) were compared in the case of normal distribution within each animal using the t-test for paired samples. The signed-rank test was used to compare non-normally distributed data. A probability of 0.05 was set as limit of statistical significance.

A possible influence of Giardia infection or the individual energy intake on the examined parameters was additionally investigated by means of a two factor analysis of variance for independent samples.

## 3. Results

### 3.1. Diet

The analysed chemical composition, as well as the energy content of the diet, is shown in Table 1.

The individual feed intake ranged from 500 to 750 g of the complete feed daily. All dogs consumed 100% of the documented amount of food. The mean daily energy intake is shown in Table 2.

### 3.2. Parameters

#### 3.2.1. Temperature

The indoor temperature measured close to the ground fluctuated between 7.5 and 13.6 °C; the temperature measured close to the surface of the doghouse roof in the exterior area was between 4.6 and 13.1 °C. In the area of the outdoor enclosure, the temperature was between 3.1 and 8.2 °C. Mean temperatures were documented at 11.2 °C (indoors), 9.1 °C (exterior), and 3 °C (outdoor area). The overall average temperature was 7.8 °C.

#### 3.2.2. Body Constitution

The body weight on day 1 was between 18.3 and 26.6 kg and developed to 19.0 to 26.8 kg by day 28. On average, an increase per dog of 1.3 kg body weight was recorded within 28 days. The shoulder height changed from 53 to 59 cm on day 1 to 53 to 60 cm on day 28. In average, the shoulder height per dog increased by 0.5 cm. Measurements of the thickness of the SCF showed values of 1.4 to 3.4 mm on day 1 and 1.4 to 2.9 mm on day 28. Changes in SCF thickness from −2 to +1.2 mm were detected in the individual animal. The thickness of the MSCDL was measured and the data showed values of 10.3 to 21.9 mm on day 1 and 13.7 to 23.2 mm on day 28. During the test period, the thickness of the SCF decreased by 0.44 mm on average, and the thickness of the measured muscle increased by 1.7 mm. The differences of the paired observations (shoulder height, body weight, and thickness of MSCDL and SCF between the first and last day of the experiment) were significant at a probability of 0.05 (Table 3).

#### 3.2.3. Physical Activity

In the cases when high activity level was documented, the dogs were each worked for an average of 24 min that day. Analogously medium activity was performed for an average of 27 min each and low activity was performed over 90 min. The number of single activity levels was documented. With the help of the total number of documented activities within 28 days of all 20 dogs, as well as the averaged time spent in activity, the time of each dog spent on average, in activity per day, could be calculated and stated in Table 4.

## 4. Discussion

Twenty intact military working dog candidates of the same breed and age (Belgian Shepherd Dogs var. Malinois, 12 months old) were kept and trained under standardized methods in central German winter temperatures. Energy intake, in connection with changes of different body condition parameters, were evaluated over 28 days.

The initial assessment of body measurements on day 1 as basis for comparative presentation showed a physically homogeneous group. During the course of the study, the average body weight increased from 22.1 kg to 23.4 kg. Hawthorne et al. published data on dogs in similar expected weight classes in adulthood (Labrador Retrievers) describing that these dogs gained body weight more slowly in advanced adolescence and had reached 99% of expected adult weight by 52 (SD ± 0.79) weeks of age [11]. It is concluded that the body weight gain of the dogs observed in the present study is only, to a small extent, due to age-related body growth, but rather to other influences, such as adaptive responses to physical activity. The increase in shoulder height of 0.5 cm corresponds to the growth pattern described by Hawthorne et al. and, in contrast to body weight, is due solely to age-related body growth.

At the time of data acquisition, the participating dogs were at the stage of their pre-training for their following specialized work. The specific service-oriented activities, such as odour detection, obedience training, and protection work should be assigned as strenuous activity. Every dog spent in average about 10 min per day (during the whole study in average 280 min) at this activity level (here categorized as moderate and high). As expected, the dogs experienced an adjustment of their physical constitution through continuously increased training in the areas of odour detection, obedience training, and protection work, with medium and high activity levels. This was recorded by documenting the development of SCF and MSCDL. The results show a significant decrease in SCF as well as a significant increase in MSCDL thickness. Physical exertion leads to muscle fibre hypertrophy in the muscle area, which is in turn physiologically associated with increased energy requirements [1] and an increase in body weight [1]. In addition, the physiological body growth at the age of 12 months, as written above, has only little influence on the expression of body weight. It could be concluded that the increased thickness of the MSCDL is due to adaptive reactions to recurrent physical training. Computer tomography (CT), magnetic resonance imaging (MRI), and dual-energy X-ray absorptiometry (DEXA) are described as methods for objective and reliable measurement of muscle mass, but with limitations, such as technique used in dogs, like repeated ionizing radiation exposure, high costs, limited access to equipment, and requirement for pharmacological immobilization with CT and MRI [12,13]. A clinically feasible ultrasound as a method to detect changes in muscle mass was chosen as an alternative in the present study to measure muscle thickness, because it was already performed in dogs with promising results and had good correlations between results of MRI- and CT-based measurements [13,14,15]. Factors limiting the reproducibility of the data obtained via ultrasonographic muscle measurements were reduced to a minimum. A single veterinarian, experienced in ultrasound examinations (standardized pressure), examined and collected the data. The chosen location (lumbar epaxial) was evaluated before in ultrasonographic muscle thickness measurements in the canine [15,16]. Fixed anatomical reference points ensured measuring at the same location. Additionally, the shaved area of 4 × 2 cm^2^ at the described location was still visible in all dogs after 28 days.

During the weekly general examinations, the dogs considered were a healthy group of 20 young dogs of the same breed. At the beginning of the study, all 20 animals were free of endoparasitic infections. At the end of the study, infection with Giardia spp. was detected in three dogs, although the time of infection after day 1 cannot be dated further. The regularly collected documentation of the individual body parameters did not show any outstanding deviations from the comparative values of the other study participants in the 3 affected dogs, and with *n* = 3 out of 20, the incidence with Giardia spp. infected animals within the population under consideration is 15%, which is below the incidence of 18.1% that is recorded for the German total dog population [17]. Statistical analysis revealed that no particular influence of the factor Giardia spp. infection on physical condition was found.

Data available in literature regarding the average energy requirement of dogs differ in some cases considerably (Table 5). These differences are based on the influence of various factors, such as breed, age, activity level, and ambient temperature [1]. For example, the National Research Council describes the energy requirement for dogs as 95 to 200 kcal/kg BW^0.75^ [1]. The lower value describes the energy requirements of inactive pet dogs without special physical activity and the higher value describes, for example, the energy requirements of active pet Great Danes kept under laboratory conditions. Under laboratory conditions in kennels or active pet dogs (environmental conditions: dogs in a domestic environment with strong stimulation and ample opportunity for exercise or multi-dog household, or with large exercise areas) have an average energy requirement of 130 kcal/kg BW^0.75^. Bermingham et al. described in a meta-analysis after evaluation of 29 publications an average energy requirement of 87.5 to 198.1 kcal/kg BW^0.75^ [4]. Included in this analysis were 713 dogs of various breeds, sizes, weight classes, and activity levels. The authors were able to demonstrate differences in energy requirements between dogs kept at home and the total population under consideration, which also included racing, hunting, and working dogs. Pet dogs had the lowest energy requirement, 124.1 kcal/kg BW^0.75^. For working dogs, an average energy requirement of 157.1 to 220.1 kcal/kg BW^0.75^ was determined, for hunting dogs 127.1 to 202.5 kcal/kg BW^0.75^ and for racing dogs 172.3 to 233.5 kcal/kg BW^0.75^. For hunting dogs, the NRC indicates a requirement of 240 kcal/kg BW^0.75^ [1]. There was no indication of ambient temperature during this data collection. Mullis et al. examined a group of 20 adult odour detection, explosive detection, and human detection dogs with regard to their energy consumption during their training on duty and found a requirement of 136 kcal/kg BW^0.75^ at an average ambient temperatures of 16 to 27 °C [6]. In addition, sled dogs were studied at an ambient temperature of −20 degrees Celsius, whose energy requirement at rest is given as 215 kcal/kg BW^0.75^ [18].

The data of the 20 Belgian Shepherd dogs in the present study showed an average energy supply of 244 kcal/kg BW^0.75^, which is comparable to that of hunting dogs and higher than described for working dogs investigated so far. Influencing factors, such as physiological growth and activity level, have already been considered, and in the present study the effect of growth was found to be less than that of activity level. In addition to the above-mentioned influences, the influence of being kept under ambient temperatures below the thermoneutral zone (TNZ) should also be discussed in connection with the available data. The effect of ambient temperatures below the TNZ during different activity levels on energy requirement is a frequent complication in scientific studies, as they are competing factors that increase energy requirement, which cannot be assessed separately [1]. The data acquisition of the present study took place in ambient temperatures between 3 °C and 13.6 °C and averaged about 8 °C. As homoiothermal animals, dogs increase their heat production when staying in temperatures outside their TNZ to ensure the maintenance of their internal body temperature. The lower limit of the TNZ is called lower critical temperature, and is described by the National Research Council for adult dogs as 20 °C to 25 °C. Breed-specific deviations may occur, the lower critical temperature is lower for breeds with dense fur than for breeds with less dense fur. The habituation is also a strong influencing factor. For example, dogs accustomed to outdoor kennel housing show less differences in energy requirements after being moved to colder ambient temperatures compared to dogs kept indoors [1]. To compare the data obtained in this study, studies with similar environmental factors were regarded. An increased energy requirement due to low ambient temperatures was demonstrated for Great Danes kept in kennels. In winter, compared to summer temperatures at 25 °C temperature difference, they required 70 kcal ME/kg BW^0.75^ per day more, which corresponds to an increase in energy requirement of 3 kcal/kg BW^0.75^/°C [19]. Durrer and Hannon came to similar conclusions when they looked at Beagles and Huskies at ambient temperatures of 14 and −20 °C respectively [20]. Blaza et al. described a 25% increase in daily feed intake in Labrador Retrievers when the animals were moved from 15 °C to 8 °C ambient temperature [21].

According to the data of NRC and Zentek et al. [1,19], in the present study, 378 kcal per day and per dog are arithmetically allotted to the increased energy requirement of the dogs due to their stay in ambient temperatures below the thermoneutral zone at 8 °C (12 °C less than TNZ of 20 °C, 3 kcal/kg BW^0.75^/°C increase in ambient temperature). The daily energy intake of 2546 kcal per dog or 244 kcal/kg BW^0.75^ during a stay in ambient temperatures of 8 °C would thus correspond to a daily energy requirement of 2168 kcal or 206 kcal/kg BW^0.75^ during a stay in ambient temperatures in the area of the TNZ at 20 °C.

This would correspond to the energy requirement of Great Danes kept under laboratory conditions and active or in outdoor kennels in summer [1,19]. For the dogs considered in this study, an energy requirement of 244 kcal/kg BW^0.75^ was evaluated, but this increase should not be attributed solely to the effect of ambient temperature. The factors mentioned, such as activity levels and, even if only to a minor extent, age-related growth, should also be mentioned here. The dogs gained in average 1.3 kg weight during the experiment. According to [22], the energy contained in 1.3 kg weight gain (added tissue) of young dogs can be estimated as being composed of 26.9% crude fat and 22.5% crude protein; therefore, containing 19.8 MJ (1.524 MJ/100 g gain; 24 kJ/g protein and 39 kJ/g fat). This mean that about 0.7 MJ per day might be attributed to weight gain and should be subtracted from MER. It should also be noted that the dogs observed developed a temperature-adapted winter coat due to habituation. This is more dense and longer than in the breeds considered in the comparative regarded studies (Great Danes and Beagles), so that the energy expenditure for heat retention of the dogs considered in the present study can be estimated to be less than in the studies indicated. This in turn suggests a stronger influence by the activity level.

In order to secure the data for MWDs in Germany, it would be desirable that the study design carried out here be repeated with changes in single factors, such as climate, age of the dogs, or, also, activity levels. The standardized husbandry and training of the dogs allows, by changing individual factors during future studies, to determine the influence of the focused individual factor, which usually competes with other accompanying factors.

## 5. Conclusions

The data of the present study show that Belgian Shepherd Dog var. Malinois at the age of 12 months, when staying below their TNZ, and during four weeks of their pre-training as MWDs, have a daily energy consumption of, on average 244 ± 34 kcal/kg BW^0.75^. Under this energy supply, the still growing dogs maintained their body weight, or even improved constitution, and were fully able to exercise. This is approximately 1.5 times higher than the stated energy requirements in international comparative literature for adult working dogs, odour detection, explosive detection, and human detection dogs, and approximately 2 to 2.5 times higher than that reported for pet dogs kept at home. The observed energy intake corresponds to the energy requirements stated for hunting dogs.

The main factors influencing the increase in energy consumption in almost adult MWDs might be the ambient temperature below the thermoneutral zone and the activity level, the differentiation to which extent must be ensured by further studies under similar standardized methods with one varying factor.

## Figures and Tables

**Table 1:** Chemical composition and energy content of the diet.

		Diet
**Dry matter (DM)**	g/kg as fed	961
**Crude ash**	g/kg DM	65.1
**Crude fibre**		16.1
**Crude protein**		293
**Crude fat**		164
**N-free extracts**		463
**ME ^1^**	kcal ME/kg as fed	4121

^1^ Metabolizable energy (ME) content of the diet was estimated in accordance with [1].

**Table 2:** Mean body weight and daily feed and energy intake of 20 dogs from days 1 to 28.

Mean Body Weight (kg)	Mean Daily Feed Intake (g)	Mean Daily Energy Intake (kcal)
22.9 ± 1.92	618 ± 90	2546 ± 371

Over the test period, daily energy intake amounted to, for each dog, an average of 112 ± 16 kcal/kg BW or 244 ± 34 kcal/kg BW^0.75^.

**Table 3:** Shoulder height, body weight, and thickness of subcutaneous fat tissue (SCF), Musculus sacrocaudalis dorsalis lateralis (MSCDL) on days 1 and 28, and their difference (mean value ±SD).

Parameter	Day 1	Day 28	Difference	*p*-Value
**Shoulder height (cm)**	56.6 ± 1.82	57.1 ± 1.96	0.5 ± 0.51	0.0020
**Body weight (kg)**	22.1 ± 2.09	23.4 ± 2.05	1.3 ± 1.13	<0.001
**SCF (mm)**	2.34 ± 0.64	1.90 ± 0.47	−0.44 ± 0.86	0.0331
**MSCDL (mm)**	15.4 ± 3.06	17.1 ± 2.53	1.7 ± 1.8	0.0005

**Table 4:** Time spent at different activity levels and total number of activities carried out in 28 days by all 20 dogs.

Activity Level	Average Time Spent Per Activity (Min)	Total Number of Activities Carried Out in 28 Days by 20 Dogs (*n*)	Time of Each Dog Spent in Average in Activity Per Day (Min)
**High activity**	24	95	4
**Medium activity**	27	114	5.5
**Low activity**	90	287	46

**Table 5:** Energy requirements and supply of dogs in relation to activity and ambient temperature.

Reference	*n*	Specification, Activity	Ambient Temperature (°C)	Obtained Energy (kcal/kg BW^0.75^)
NRC [1]		Hunting dogs		240 (200–280)
		Working Collies		184 (80–380)
		Racing Greyhounds		140 (120–160)
		Inactive pet dogs ^a^		95
		Active pet dogs ^b^		130
	-	Active pet Great Danes		200
Thes et al. [2]	586	Privately owned pet dogs		98 ± 29
Dinallo et al. [5]	238	Agility dogs		106 ± 33
Bermingham et al. [4]	713	Average of all included dogs		88–198
	216	Pet husbandry		124
	56	Working dogs		157–220
	14	Hunting dogs		127–203
	46	Racing dogs		172–234
Mullis et al. [6]	20	Odour, explosive, and human detection working dogs while training	20	136
Hill [18]		Sled dogs in rest	−20	215
Zentek and Meyer [19]	7	Great Danes in outdoor kennels	summer	≈200
	7	Great Danes in indoor kennels	winter	≈250
Durrer and Hannon [20]	5	Beagles in outdoor husbandry, temperature reduction of 34 °C	16 to −18	+77%
	5	Huskies in outdoor husbandry, temperature reduction of 34 °C	16 to −18	+69%
Blaza [21]	4	Labrador retriever, temperature reduction of 7 °C	15 to 8	+25%
present study	20	Young MWDs in pre-training (12th month of life)	8	244

^a^ Dogs kept in a domestic environment with little stimulation and little opportunity to exercise. ^b^ Dogs kept in a domestic environment with strong stimulation and ample opportunity to exercise, such as multiple-dog household in the country, or with a large yard. NRC (National Research Council). MWDs (Military working dogs).
